# Molecular epidemiology and virulence of goose astroviruses genotype-2 with different internal gene sequences

**DOI:** 10.3389/fmicb.2023.1301861

**Published:** 2023-12-07

**Authors:** Linhua Xu, Bowen Jiang, Yao Cheng, Zhenjie Gao, Yu He, Zhen Wu, Mingshu Wang, Renyong Jia, Dekang Zhu, Mafeng Liu, Xinxin Zhao, Qiao Yang, Ying Wu, Shaqiu Zhang, Juan Huang, Xumin Ou, Qun Gao, Di Sun, Anchun Cheng, Shun Chen

**Affiliations:** ^1^Research Center of Avian Disease, College of Veterinary Medicine, Sichuan Agricultural University, Chengdu, Sichuan, China; ^2^Key Laboratory of Animal Disease and Human Health of Sichuan Province, Sichuan Agricultural University, Chengdu, Sichuan, China; ^3^Engineering Research Center of Southwest Animal Disease Prevention and Control Technology, Ministry of Education of the People's Republic of China, Chengdu, China

**Keywords:** goose astrovirus, genotype-2, internal gene, molecular epidemiology, virulence

## Abstract

Goose astrovirus (GAstV) is a small, non-enveloped, single-stranded, positive-sense RNA virus. GAstV has rapidly spread across various regions in China since 2016. In Sichuan, out of 113 samples were collected from goose diseases between 2019 and 2022, 97 were positive for GAstV through PCR testing. Remarkably, over the past three years, GAstV outbreak in Sichuan has accounted for an astonishing 85.8% of all goose-origin viruses. Among these cases, 63.9% had single GAstV infections, 29.9% had dual infections, and 6.2% had quadruple infections. To comprehend the variations in virulence among distinct strains of GAstV. 12 representative strains of single GAstV infections were isolated. These strains exhibited distinct characteristics, such as prominent white urate depositions in organs and joints, as well as extensive tissues phagocytosis in major target organs’ tissues. The conserved ORF1b genes and the variable ORF2 genes of these representative GAstV strains were sequenced, enabling the establishment of phylogenetic trees for GAstV. All GAstV strains were identified as belonging to genotype-2 with varying internal gene sequences. Experiments were conducted on GAstV genotype-2, both *in vivo* and *in vitro*, revealed significant variations in pathogenicity and virulence across susceptible cells, embryos, and goslings. This comprehensive study enhances researchers’ understanding of the transmission characteristics and virulence of GAstV genotype-2, aiding in a better comprehension of their molecular epidemiology and pathogenic mechanism.

## 1 Introduction

Goose astrovirus is a small, non-enveloped, positive-sense RNA virus (Zhu and Sun, 2022; Zhang F. et al., 2022; Zhang X. et al., 2022). Its genome consists of a 5′-untranslated region (UTR), three open reading frames (ORF1a, ORF1b, and ORF2), a 3’-UTR, and a poly(A) tail (Yang et al., 2018; Ding et al., 2021). The ORF1a genes and ORF1b genes encode non-structural viral proteins (NSPs) that contain elements such as transmembrane domains (TMs), serine protease motifs, zinc protein models, nuclear localization signals, and RNA-dependent RNA polymerases (RdRps) (Wei et al., 2020b). Conversely, the ORF2-encoded viral capsid protein (CP) exhibits substantial genomic diversity, with conserved N-terminal segments and variable C-terminal regions (Chen Q. et al., 2020). The core antigenic components of GAstV are the capsid proteins (Ren et al., 2020). Adhering to the classification guidelines set by the International Committee on Taxonomy of Viruses (ICTV), classification is often based on the p-distance (p-dist) calculated between the full amino acid sequences of the hosts and the structural protein (Zhang M. et al., 2022). Members of the same genotypes species typically exhibit p-dist values >75% within their complete capsid protein sequences (Wang H. et al., 2022). Significantly, the p-dists between GAstV genotype-1 and GAstV genotype-2 stand at 0.609 ± 0.021. This value is akin to the p-dist observed between distinct avian astrovirus (ranging from 0.576 to 0.741), highlighting the presence of two distinct astrovirus variants in goslings (Fei et al., 2022). In accordance with this viral classification criterion, GAstV can be divided into two distinct genotypes: GAstV genotype-1 and GAstV genotype-2. Moreover, there are significant differences in strains characteristics between these two genotypes (Niu et al., 2018; Wang et al., 2021; Fu et al., 2022).

GAstV has erupted and spread extensively across various regions of China, emerging as a grave threat to the goose breeding industry in recent years (Xu et al., 2019; Yin et al., 2021; Zhu et al., 2022). After their initial outbreaks, GAstV rapidly spreads throughout various regions in China (Ji et al., 2020). In the most recent survey of viruses originating from geese in China, a staggering 50.84% of these viruses were identified as GAstV (Yuan et al., 2019; Yin et al., 2020; He et al., 2022; Yi et al., 2022). The infection rates of GAstV in clinical samples from six northern provinces of China reached 81.5%, resulting in substantial economic losses for the goose industry (Liu et al., 2020). Infected goslings typically exhibit symptoms such as depression, anorexia, accompanied by gray and cloudy eyelids (Zhang Q. et al., 2018; Liu et al., 2019; Yang et al., 2021; Wang A. et al., 2022). Notably, key pathological characteristics of GAstV infection are the extensive depositions of urate within the viscera and joints of affected goslings, leading to inhibited growth (Wu et al., 2020). The fatality rate associated with GAstV infection generally ranges between 20 and 50%. Moreover, GAstV has the potential for vertical transmission from geese to goslings (Wei et al., 2020a). The remarkable genetic diversity and recombination capabilities of GAstV underscore its ability to trigger diverse diseases across various hosts, even crossing species barriers to infect Pekin ducks (Wei et al., 2020b), Cherry Valley ducks (Chen H. et al., 2020), Moscow ducks (Chen et al., 2021), and other hosts. GAstV poses a significant threat to the waterfowl industry’s development. The susceptibility of GAstV to changes in virulence is intricately linked to the structure of its single-stranded genome (Xu et al., 2019; He et al., 2020), with the highly variable nature of this genome being a probable reason for its efficacy in host-to-host transmission (Cui et al., 2022).

In a concerted effort to delve deeper into transmission characteristics and pathogenicity of distinct GAstV genotype-2, this study systematically collected 113 samples from infected goslings across various farms in Sichuan and isolated 12 single-infected GAstV strains from them. By analyzing the strains’ pathological characteristics, phylogenetics, distributions, host adaptability, virulence, as well as pathogenicity. A comprehensive understanding was achieved regarding molecular epidemiology and characteristics of GAstV genotype-2. As far as our knowledge extends, this research represents the inaugural exploration into the virulence and host adaptability of GAstV genotype-2. It stands as the first systematic documentation, furnishing critical insights into the myriad aspects of GAstV genotype-2 within this specific locale. The findings from this research contribute to the enhancement of researchers’ comprehension on epidemiology and pathogenesis of GAstV genotype-2.

## 2 Materials and methods

### 2.1 Cell lines, embryos, and viruses

10-days-old goose embryos and duck embryos were sourced from the Ya’an Animal Breeding Base of Sichuan Agricultural University. 3-day-old Tianfu meat goslings were also obtained from the same breeding base. LMH cells were cultured in Dulbecco’s modified Eagle’s medium Nutrient Mixture F-12 (DMEM-F12) supplemented with 10% fetal bovine serum (FBS), both of which were provided by Gibco (Shanghai, China). Twelve single-infected strains of GAstV were successfully isolated from deceased goslings within the Sichuan region in Southwest China.

### 2.2 Samples collection

Several goose farms in Sichuan have reported a recurring issue of goslings infected with GAstV displaying symptoms of depression and anorexia in recent years. Significantly, some of these infected goslings have developed gray and cloudy eyelids, coupled with inhibited growth. GAstV’s capacity to replicate within various tissues has been observed, encompassing organs like the hearts, livers, spleens, kidneys, and intestines. Upon autopsy, significant urate depositions have been noted within both the viscera and joints of the afflicted goslings. To investigate further, PCR or RT-PCR analyses were conducted on these diseased goose samples, all of which had succumbed to non-environmental factors. For this study, the 12 selected strains of GAstV were thoroughly screened for the presence of other viruses, including but not limited to gosling plague viruses, tembusu viruses, goose reoviruses, adenoviruses, and avian influenza viruses. The results indicated the absence of these viruses in the examined GAstV strains, highlighting their specificity and isolation from other potential pathogens.

### 2.3 Viruses DNA/RNA genomes extraction

To investigate the potential causative pathogens or pathogens behind this disease outbreaks, viruses obtained from an intestinal and liver homogenate were subjected to blind passages in 10-day-old goose embryos via allantoic cavity injection. Following infection, the allantoic fluids from the affected embryos were harvested and subsequently centrifuged at 7,500 g/min for a duration of 20 min to eliminate cells debris. The resulting supernatant was collected and subjected to further ultracentrifugation at 9,000 g/min for 2 h. The resultant pellet was resuspended in PBS and utilized for the extraction of viral DNA/RNA. Distinct viral DNA and RNA components were extracted separately utilizing the TIANamp Virus DNA/RNA Fast Kit (TIANGEN, Beijing, China), following the manufacturer’s stipulated protocols. The concentration of the extracted RNA was determined utilizing the Nanodrop 2000.

### 2.4 Virus identification and phylogenetic analysis

The isolation of total RNA from cells lysates, allantoic fluids, and tissue homogenates was executed using the Trizol extraction method (Takara, Dalian, China). Reverse transcription fluorescence quantitative PCR (RT-qPCR) was conducted on the Bio-Rad (Hurcules, CA). For the detection of viral RNA in infected samples, complementary DNA (cDNA) was first synthesized using the HiScript QRT SuperMix (Vazyme) as per the manufacturer’s instructions for RT-PCR. Subsequently, real-time RT-PCR was conducted using the 2 × Taq SYBR Green qPCR mix (Innovagene, Changsha, China). The PCR products originating from the GAstV ORF1b genes were employed to establish a standard curve, thereby facilitating the absolute quantification of GAstV genome copies via the obtained Ct values. The quantified viral genome values were normalized against the absolute genome copies of the GAstV ORF1b genes, and the outcomes were expressed as GAstV genome copies per milligram (mg) of total RNA. To quantify viral load within infected tissues and allantoic fluids, the viral load was reported in terms of absolute genome copies per microliter (μL) of viral fluids. This quantification was derived from the standard curve generated using the product of the GAstV ORF1b genes. Primers used for RT-PCR or RT-qPCR in this study (Table 1). Primers: RT-PCR-GAstV-F/R 1–4 stand for the primers used for segmented sequencing of different strains of GAstV. Phylogenetic analysis was carried out using ORF1b and ORF2 internal sequences of GAstV, multiple alignments were constructed using the ClustalW method available within the MEGA 7.0 software. For the construction of phylogenetic trees of each gene segment for the twelve goose astroviruses, MEGA 7.0 with the distance-based neighbor-joining method was applied.

**Table 1:** Primers used for RT-PCR or RT-qPCR in this study.

Primer name	Primer sequence (5′ → 3′)	Product size (bp)
RT-PCR-GAstV-F1	GAGAATAAGAAGAACATTTT	1815
RT-PCR-GAstV-R1	AGAAGTCGGGCCCGACCTC	
RT-PCR-GAstV-F2	TCTGGGGTAAATTGGTTTC	844
RT-PCR-GAstV-R2	TCACGTAAATGACAAAAGTT	
RT-PCR-GAstV-F3	AGTGCATTTACTGTTTTCAA	1,500
RT-PCR-GAstV-R3	TCGGCGTGGCCGCGGCTGCT	
RT-PCR-GAstV-F4	TGGTGGTGTTTTCTCAAAAATGA	788
RT-PCR-GAstV-R4	ACATTGGGAACTCCAACAAA	
RT-PCR-GPV-F	GGGTGCCGATGGAGTGGG	661
RT-PCR-GPV-R	GAGCCTGTCTAAGTCCTGTG	
RT-PCR-ADV-F	TGCGACAACTACCTGTGGAC	235
RT-PCR-ADV-R	GCGTACGGAAGTAAGCCAT	
RT-PCR-ARV-F	GTTCCATTCTGCTCCCCGG	634
RT-PCR-ARV-R	CGTCGAACACCATGTCAACC	
RT-PCR-TMUV-F	GCCACGGAATTAGCGGTTGT	401
RT-PCR-TMUV-R	TAATCCTCCATCTCAGCGGTGTAG	
RT-PCR-AIV-F	TTCTAACCGAGGTCGAAAC	229
RT-PCR-AIV-R	AAGCGTCTACGCTGCAGTCC	
PCA-GAstV-ORF1b-F	CATCATTTTGGCAAAGAATTCGCCAC CATGGGCAGGATGATATTATTGAGTG	4,880
PCA-GAstV-ORF1b-R	TTGGCAGAGGGAAAAAGATCT CTAGGAGCATATTCATCTTGTTG	
RT-qPCR-GAstV-F	GGCAGGATGATATTATTGAGTG	160
RT-qPCR-GAstV-R	GGAGCATATTCATCTTGTTG	

### 2.5 Virus growth kinetics in susceptible cells and embryos

In brief, the experimental process involved the following steps. LMH cells were introduced into a 12-well plate, with 1 mL of culture medium added to each well. The LMH cells were divided into two groups (the infected group and the control group). Each group comprised three separate cells wells, serving as biological replicates. The infected group contained LMH cells inoculated with each of the 12 GAstV genotype-2, while the control group remained untreated. After a 48-h period of GAstV infection, molecular techniques like RT-PCR and RT-qPCR were utilized. RT-PCR and RT-qPCR were applied to detect GAstV infection and quantify the respective GAstV genome copies. For the *in vivo* and *in vitro* aspect of the study, 12 GAstV genotype-2 were injected separately into goose embryos and duck embryos. Each strain was introduced into three embryos, serving as biological replicates. A control group consisted of embryos injected with an equal volume of phosphate-buffered saline (PBS). The viral dose for each strain was set at 2 × 10^4^ TCID_50_. Throughout the experiment, daily checks were conducted on embryos survival. In instance of embryos mortality, allantoic fluids were promptly collected for subsequent RT-PCR and RT-qPCR analysis. Alternatively, if embryos remained viable, RT-qPCR was employed to track viral load for a period of seven days post infection. In summary, these procedures enabled the comprehensive assessment of GAstV responses within LMH cells and embryos, facilitating insights into infection dynamics, replication, and its impact on host organisms.

### 2.6 Animal infection experiments

The animal infection experiments aimed to assess the impact of GAstV infection on goslings, focusing on factors such as mortality rate, viral load, and changes in body weight. Newly 3-day-old goslings, sourced from the Ya’an breeding base of Sichuan Agricultural University, were used for the experiment. Each gosling was injected with 5 × 10^4^ TCID_50_ of different GAstV strains. Three goslings were injected with each strain, serving as biological replicates. A control group was established, with three goslings injected with the same volume of phosphate-buffered saline (PBS). Goslings’ health and behavior were closely monitored post-infection. Goslings showing signs of not eating or drinking were euthanized. Organ tissue samples, including infected livers, spleens, kidneys, and others, were collected daily from deceased goslings. This animal infection experiments sought to provide insights into the effects of GAstV infection on goslings, offering a comprehensive assessment of mortality rates, viral load, and physiological changes in response to different GAstV strains.

### 2.7 Histology HE staining and immunohistochemical staining

A portion of the spleen, liver, heart, brain, kidney and intestine samples was fixed in buffered 10% formalin for 24 h, dehydrated in graded alcohol, embedded in paraffin wax and cut into 5-μm-thick sections. Some sections were stained with haematoxylin and eosin (H&E) using a conventional protocol. Immunohistochemical (IHC) staining was performed at the same time. Briefly, a mouse monoclonal antibody against GAstV Cap protein was diluted at 1:500. After an overnight incubation with the primary antibody at 4°C and three washes with PBST, the sections were incubated with the goat anti-mouse secondary antibody (Biotin-Streptavidin HRP Detection Systems, ZSGB-BIO, Beijing, China) for 30 min at 37°C. Finally, the sections were observed under an optical microscope (Nikon, Tokyo, Japan).

### 2.8 Viral titers detection

Viral titers were determined by the median tissue culture infectious dose 50 (TCID_50_) method in LMH cells. Viral samples were serially diluted 10-fold in DMEM, and then 100 μL dilutions of the viral sample were distributed to each of 8 wells of a 96-well plate seeded with a monolayer of LMH cells. After 120 h incubation at 37°C with 5% CO2, the presence of viruses was detected by assaying CPE using microscopy, and the viral titers were calculated according to the Reed-Muench method.

### 2.9 Quantification and statistical analysis

Data of the RT-qPCR is presented as means ± Standard Error (SEM). Student’s t-test was used to assess statistical significance, with significance defined by *p* value <0.05 (*) in GraphPad Prism 9.0 software. Statistical significance of survival was analyzed using survival curve, Log-rank (Mantel-Cox) test in GraphPad Prism 9.0 software.

### 2.10 Ethical statements

All animal experimental procedures were approved by the Institutional Animal Care and Use Committee of Sichuan Agriculture University in Sichuan, China (Protocol Permit Number: SYXK (川)2019-187).

## 3 Results

### 3.1 Pathological characteristics of the goose astrovirus isolates

**Figure 1:**
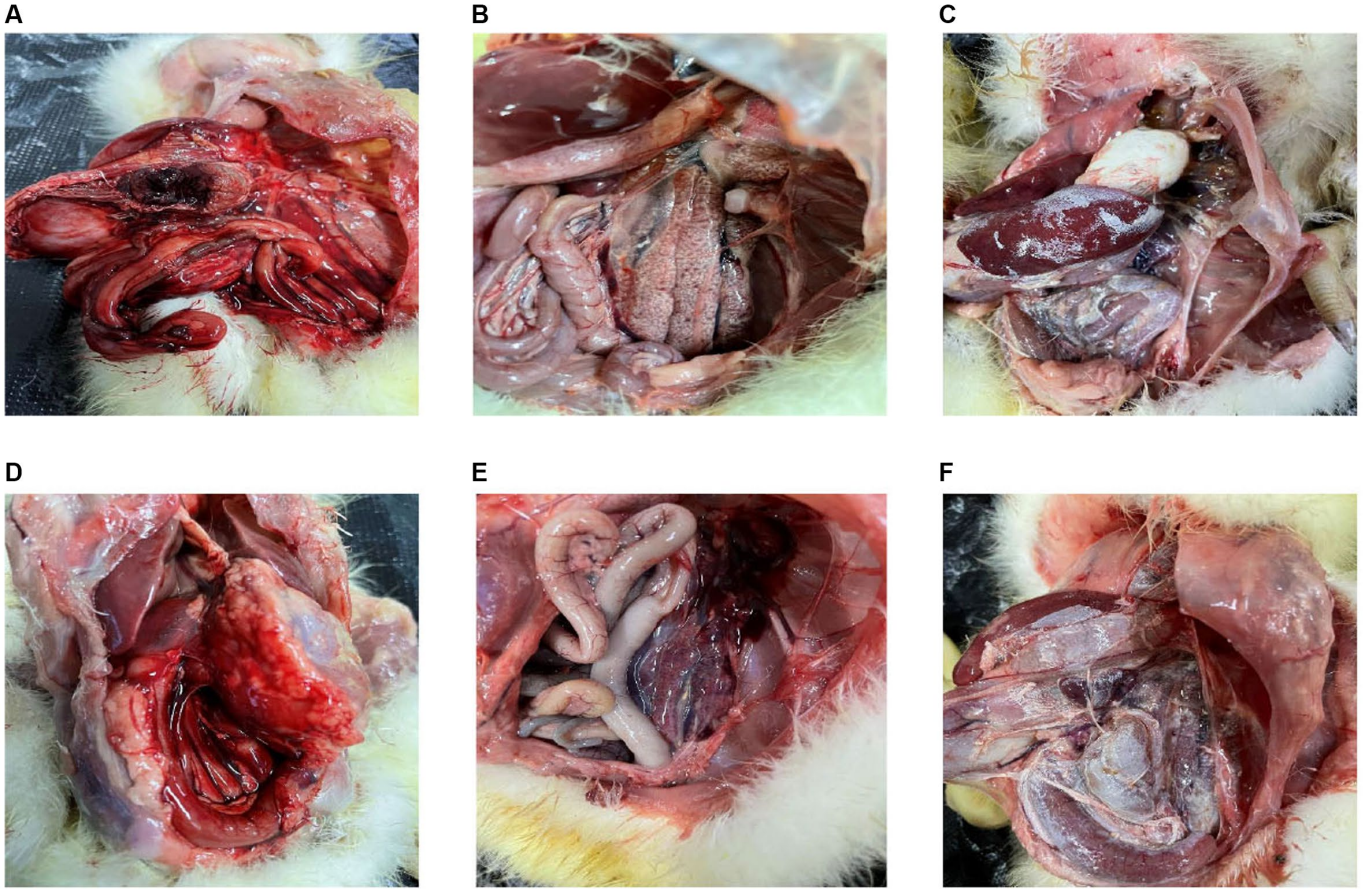
Pathologic changes in goslings infected with goose astroviruses genotype-2. **(A)** Congestion on the back of the livers. **(B)** Deposit of urate on the intestines. **(C)** Deposit of urate on the hearts. **(D)** Gastric redness and swelling. **(E)** Congestion in the kidneys. **(F)** Deposit of urate on the surface of livers.

A significant number of goslings in Sichuan within Southwest China experienced mortality between 2019 and 2022, exhibiting distinct clinical manifestations including blackened eyelids, unsteady posture, reduced appetite, and substantial weight loss. Investigation into these fatalities involved the examination of liver tissues obtained from the deceased goslings. Through the application of RT-PCR, these liver tissues were confirmed to be infected with GAstV. Upon conducting dissections on the affected goslings, a range of notable observations emerged. An examination of the infected livers revealed marked congestion on the posterior side (Figure 1A). Urate deposits were conspicuously present on both the intestines (Figure 1B) and the hearts (Figure 1C) of the infected goslings. The gastric region of the deceased goslings exhibited evident redness and swelling (Figure 1D). The kidneys of the affected goslings were characterized by significant congestion (Figure 1E). Particularly noteworthy was the presence of substantial urate salt depositions on the surface of the liver (Figure 1F). These findings collectively offer valuable insights into the pathological impact of GAstV infection on the goslings. The presence of urate deposits and organ abnormalities underscores the systemic effects and varied organs involvements associated with GAstV infection in the affected goslings.

### 3.2 Molecular epidemiology and infection characteristics of the goose astrovirus isolates

**Figure 2:**
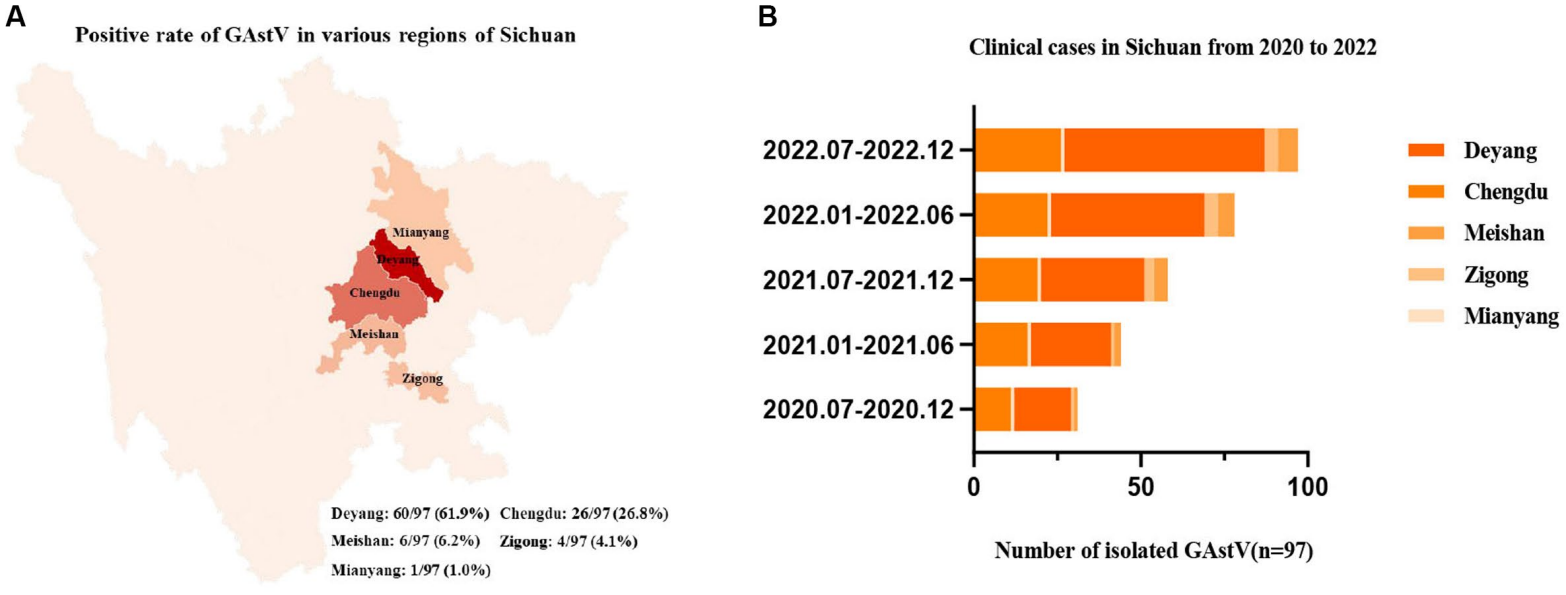
Epidemiological analysis of goose astroviruses in Sichuan within Southwest China. **(A)** Positive rates of goose astroviruses in various regions of Sichuan (*n* = 97). **(B)** Clinical case statistics of goose astroviruses from 2019 to 2022 (*n* = 97).

To gain a more comprehensive understanding of the outbreaks and prevalence of GAstV in Sichuan, a series of measures were undertaken. RT-PCR was employed to detect and differentiate various disease types, encompassing notable candidates like goose astroviruses, gosling plague viruses, tembusu viruses, goose reoviruses, adenoviruses, and avian influenza viruses. The comprehensive analysis involved 97 samples from Sichuan, China. Key observations and findings from this investigation as follows: the regions in Sichuan with the highest incidence of GAstV were identified as Deyang, Chengdu, Meishan, Zigong, and Mianyang (Figure 2A). The presence of mixed infection, particularly GAstV along with other goose-origin viruses, intensified the epidemic situation of goose-origin viruses. This complexity further highlighted the interconnectedness of various viral agents in contributing to disease outbreaks. Among the analyzed samples, 63.9% (62 cases) exhibited exclusive GAstV infection, making it the most common infection type. Meanwhile, 16.5% of cases were co-infected with gosling plague viruses, 13.4% with tembusu viruses, and 6.2% with a combination of goose reoviruses, adenoviruses, and avian influenza viruses (Figure 2B). Significantly, GAstV infection constituted a significant portion, accounting for 85.8% of all goose-origin virus samples analyzed (Table 2). This information provided valuable insights into the overarching influence of GAstV among the array of goose-origin viruses. The intricate interactions between different viral agents underscore the complex nature of the epidemic situation, thereby contributing essential reference points for effective management and control strategies These findings offer a deep comprehension on the regional dynamics of GAstV strains and their characteristics during outbreaks.

**Table 2:** Infection statistics of goose astroviruses within samples collected from farms in Sichuan.

Region	Sample	Monoinfection	Coinfection with		
			GPV	TMUV	ARV, AdV and AIV
Deyang	60	37	10	13	0
Chengdu	24	18	0	0	6
Meishan	6	0	6	0	0
Zigong	4	4	0	0	0
Mianyang	3	3	0	0	0
Sum	97	62	16	13	6
Infection rate	97/113 (85.8%)	62/97 (63.9%)	16/97 (16.5%)	13/97 (13.4%)	6/97 (2.1%)

GAstV, goose astrovirus; GPV, gosling plague virus; TMUV, tembusu virus; GRV, goose reovirus; ADV, adenovirus; AIV, avian influenza virus.

### 3.3 Genetic evolution analysis of the twelve goose astrovirus isolates

**Figure 3:**
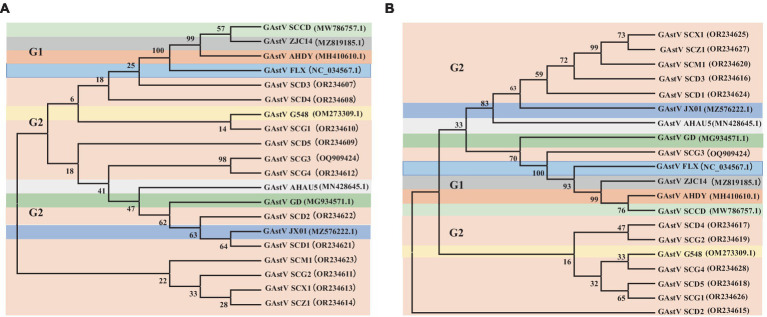
Phylogenetic analysis of goose astroviruses genotype-2. **(A)** Phylogenetic analysis of goose astroviruses genotype-2 based on ORF1b genes. **(B)** Phylogenetic analysis of goose astroviruses genotype-2 based on ORF2 genes. The trees were generated using MEGA 7.0 software and the Neighbor-joining method with 1,000 bootstrap replicates.

In the effort to analyze 12 strains of GAstV, four primer pairs were designed to sequence both the ORF1b and ORF2 genes. These sequences were subsequently aligned and compared to gather insights into their genetic characteristics. Key observations from the genetic analysis are as follows: the sequence alignment of the ORF1b genes from the 12 GAstV genotype-2, alongside four representative strains of GAstV genotype-1 and four representative strains of GAstV genotype-2, indicated the highest homology with GAstV genotype-2 (Figure 3A). Similarly, the sequence alignment of the ORF2 genes showcased the closest genetic affinity to GAstV genotype-2 (Figure 3B). In order to better analyze the homology between different strains of goose astroviruses, we compared the nucleotide sequences of different GAstV strains with the virulent strain (GAstV SCG3), and presented the detailed information of the strains in Table 3. These findings collectively underscore that there exists a single genotype of GAstV, specifically genotype-2, that prevails in the Sichuan region. This genetic uniformity provides valuable insight into the genetic makeup of the circulating GAstV strains, further informing our understanding of the viral strains prevalent in the area.

**Table 3:** Alignments with the nucleotide identities of GAstV SCG3.

Goose astrovirus genotype-2	Percent Identify (%) of amino acids (aa) to Goose astrovirus SCG3 (OQ909424)			
	Isolate Date	Isolate Region	ORF1b (GenBank accession no.)	ORF2 (GenBank accession no.)
Goose astrovirus SCD1	2021.10.18	Chengdu	98.58 (OR234621)	98.35 (OR234624)
Goose astrovirus SCD2	2021.12.14	Chengdu	98.52 (OR234622)	98.49 (OR234615)
Goose astrovirus SCD3	2021.12.28	Chengdu	97.88 (OR234607)	97.64 (OR234616)
Goose astrovirus SCG1	2021.12.30	Deyang	98.14 (OR234610)	98.11 (OR234626)
Goose astrovirus SCD4	2022.02.15	Chengdu	97.69 (OR234608)	98.58 (OR234617)
Goose astrovirus SCG2	2022.05.07	Deyang	98.90 (OR234611)	98.53 (OR234619)
Goose astrovirus SCX1	2022.04.20	Chengdu	98.71 (OR234613)	98.25 (OR234625)
Goose astrovirus SCM1	2022.05.01	Meishan	98.52 (OR234623)	98.49 (OR234620)
Goose astrovirus SCD5	2022.05.17	Chengdu	97.88 (OR234609)	98.25 (OR234618)
Goose astrovirus SCZ1	2022.05.18	Zigong	98.77 (OR234614)	98.30 (OR234627)
Goose astrovirus SCG4	2022.08.27	Deyang	98.20 (OR234612)	98.49 (OR234628)

### 3.4 Growth kinetics of the goose astroviruses in susceptible cells and embryos

**Figure 4:**
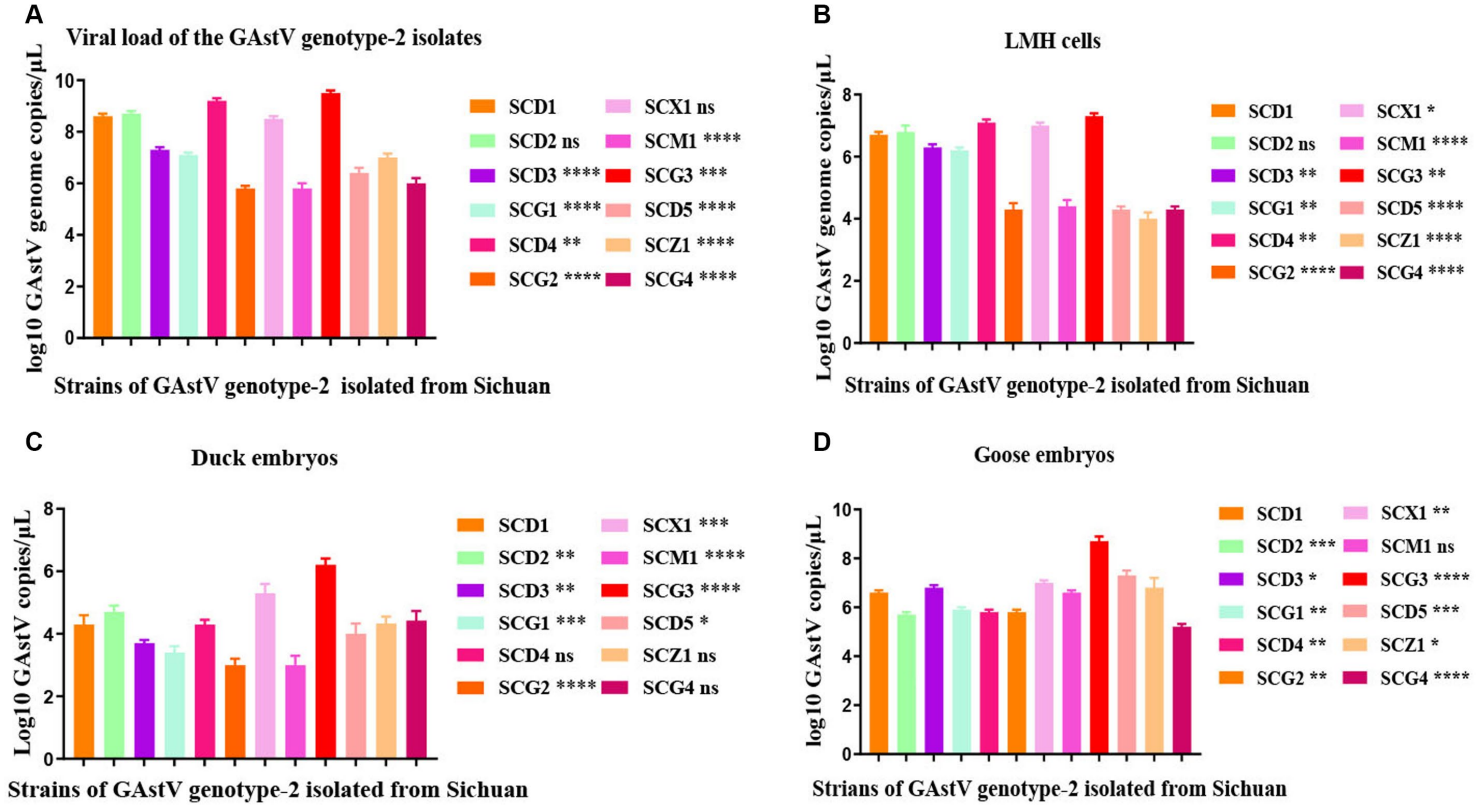
Replication kinetics of goose astroviruses genotype-2 *in vivo* and *in vitro*. **(A)** Viral load of goose astroviruses genotype-2 isolated from Sichuan. LMH cells from the experimental group (*n* = 36) were infected with 2 × 10^4^ TCID_50_ of goose astroviruses genotype-2 (each strain *n* = 3), while embryos from the control group (*n* = 3) were mock infected with equal volume of PBS. **(B)** Viral load change in LMH cells infected with goose astroviruses genotype-2. 10-day-old goose embryos and duck embryos from the experimental group (*n* = 36) were infected intravenously with 2 × 10^4^ TCID_50_ of goose astroviruses (each strain *n* = 3), while embryos from the control group (*n* = 3) were mock infected with equal volume of PBS. Statistical results one generation post infection. **(C)** Viral load change in duck embryos infected with goose astroviruses genotype-2. Statistical results one generation post infection. **(D)** Viral load change in goose embryos infected with goose astroviruses genotype-2. Statistical results one generation post infection. * Indicates statistically significant differences (ns indicates not significant, * indicates *p* ≤ 0.05, ** indicates *p* ≤ 0.01, *** indicates *p* ≤ 0.001, **** indicates *p* ≤ 0.0001).

The isolation and analysis of 12 GAstV genotype-2 from Sichuan yielded interesting findings about their behavior and pathogenicity. The 12 isolated GAstV strains exhibited varying viral load. For instance, the SCG3 strain showed a notably higher viral load, while the SCM1 strain displayed lower viral load (Figure 4A). Different GAstV strains exhibited differing sensitivities to LMH cells. Those strains with higher viral load demonstrated successful cultivation in susceptible LMH cells (Figure 4B). The sensitivity of GAstV strains to goose embryos and duck embryos varied. Among the strains, only SCX1 and SCG3 effectively replicated and propagated in goose embryos (Figure 4C). Overall, the adaptability of all GAstV strains to goose embryos exceeded their adaptability to duck embryos. Significantly, strains with higher viral load displayed enhanced adaptability to both goose embryos and duck embryos (Figure 4D). These observations collectively shed light on the diverse responses and adaptability of different GAstV strains. The variations in viral load, cells culture sensitivity, and embryos adaptability contribute to understanding of how these strains interact with different hosts.

### 3.5 Virulence of the goose astroviruses in susceptible embryos and goslings

**Figure 5:**
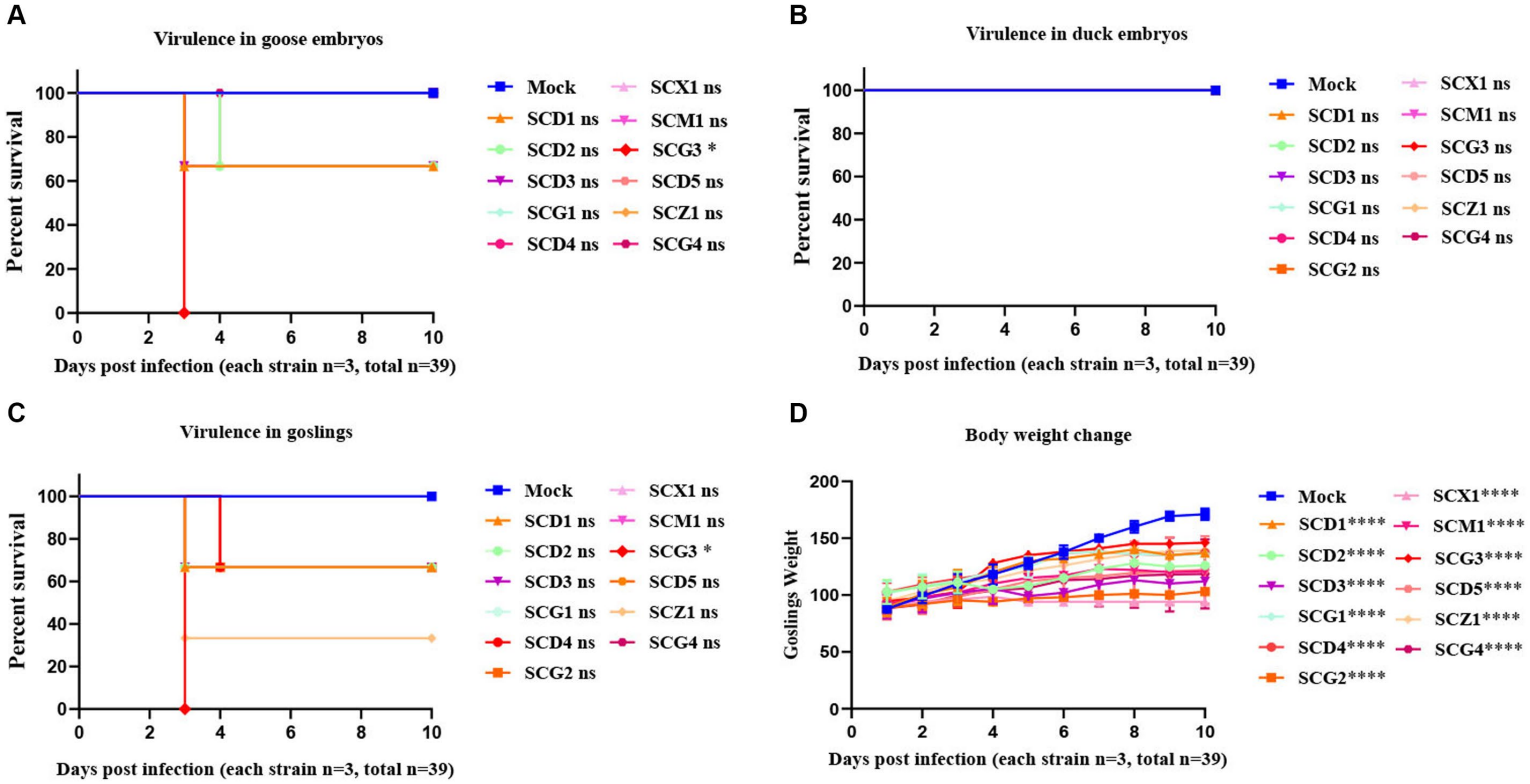
Virulence evaluation of goose astroviruses genotype-2 isolated from Sichuan. 10-day-old goose embryos and duck embryos from the experimental group (*n* = 36) were infected intravenously with 2 × 10^4^ TCID_50_ of goose astroviruses genotype-2 (each strain *n* = 3), while embryos from the control group (*n* = 3) were mock infected with equal volume of PBS. **(A)** Virulence of goose astroviruses genotype-2 in goose embryos. **(B)** Virulence of goose astroviruses genotype-2 in duck embryos. 3-day-old goslings from the experimental group (*n* = 36) were infected intravenously with goose astroviruses genotype-2 at 5 × 10^4^ TCID_50_ (each strain *n* = 3), while goslings from the control group (*n* = 3) were mock infected with equal volume of PBS. **(C)** Virulence of goose astroviruses genotype-2 in goslings. **(D)** Goslings were weighed at the indicated days post infection (dpi 1, 2, 3, 4, 5, 6, 7, 8, 9, 10) with goose astroviruses genotype-2. * Indicates statistically significant differences (ns indicates not significant, * indicates *p* ≤ 0.05, **** indicates *p* ≤ 0.0001).

The investigation into GAstV strains in relation to their virulence and impact on different hosts led to noteworthy observations. The virulence of GAstV strains differed, affecting the fatality rate of goose embryos. Strains with higher viral load demonstrated the capability to induce higher embryos mortality, with strains with elevated viral load causing easier fatalities (Figure 5A). Interestingly, none of the GAstV strains were found to be lethal to duck embryos (Figure 5B). The majority of GAstV strains exhibited the ability to infect goslings effectively. Many strains resulted in high levels of viral load detected within the target organs of the goslings. Notably virulent strains, such as SCG3, were capable of causing the death of all three infected goslings (Figure 5C). Each GAstV strain displayed varying degrees of impacts on gosling weight. Overall, all strains significantly inhibited the growth of gosling weight (Figure 5D). These findings illustrate the diverse impacts of GAstV strains on different hosts and their varying levels of virulence. The experimentally observed effects on embryos survival, gosling infection, and growth inhibition contribute to our comprehension on the range of outcomes associated with different GAstV strains.

### 3.6 Transmissibility and pathogenicity of the representative goose astrovirus in goslings

**Figure 6:**
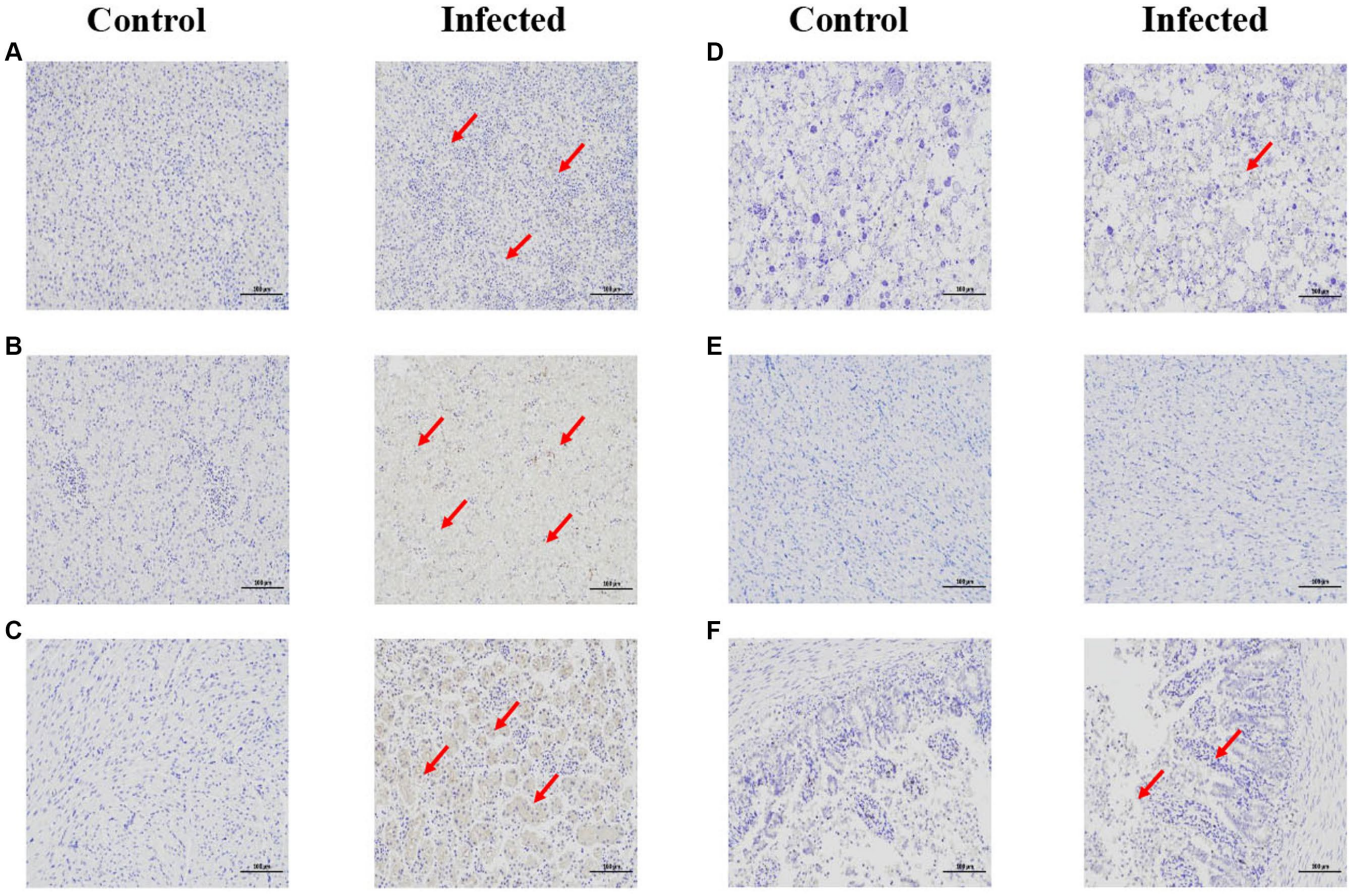
Immunohistochemical staining of infected and control tissues. 3-day-old goslings from the experimental group (*n* = 36) were infected intravenously with goose astroviruses genotype-2 at 5 × 10^4^ TCID_50_ (each strain *n* = 3), while goslings from the control group (*n* = 3) were mock infected with equal volume of PBS. **(A)** Spleen. **(B)** Liver. **(C)** Kidney. **(D)** Heart. **(E)** Brain. **(F)** Intestine. Goose astroviruses distribution in the dead gosling tissues post infection with goose astroviruses genotype-2 evaluated via an IHC analysis. Red arrows indicate positive signals. Scale bar, 100 μm.

**Figure 7:**
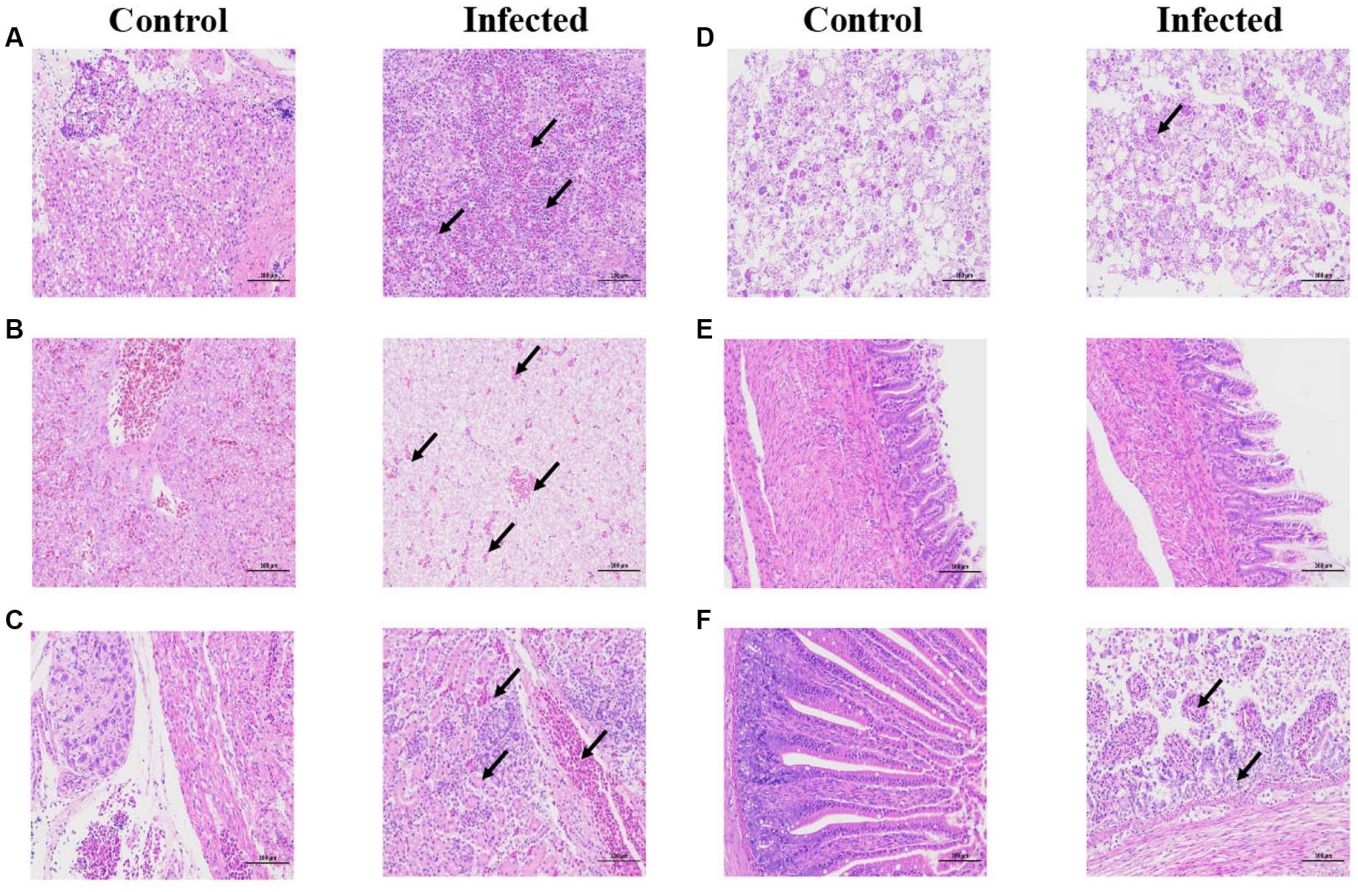
Histopathological staining of infected and control tissues. 3-day-old goslings from the experimental group (*n* = 36) were infected intravenously with goose astroviruses genotype-2 at 5 × 10^4^ TCID_50_ (each strain *n* = 3), while goslings from the control group (*n* = 3) were mock infected with equal volume of PBS. **(A)** Spleen. **(B)** Liver. **(C)** Kidney. **(D)** Heart. **(E)** Brain. **(F)** Intestine. Black arrows indicate different pathological characteristics. Scale bar, 100 μm.

Through immunohistochemistry experiments, it can be found that the positive signal in the liver is the strongest, while the positive signal in intestine, heart, and brain is very weak (red arrow). indicating that the antigen of GAstV is mainly distributed in liver, followed by kidney, spleen, intestine, heart and brain (Figures 6A–F), According to the grading of positive cell rate, the average positive rate of liver, kidney and spleen reach over 51% (level 3), which can indicate that the antigen of GAstV is enriched in liver, kidney and spleen. Through pathological analysis, a small number of scattered lymphocytes show necrosis with fragmented nuclei, along with a mild infiltration of neutrophils (Figure 7A). In the liver tissue, there is diffuse hepatocellular steatosis with varying-sized round vacuoles in the cytoplasm; there is mild congestion and dilation in multifocal liver sinusoids; a significant amount of central vein congestion is observed; occasional presence of numerous white blood cells in central veins (Figure 7B). In the kidney tissue, there is widespread thickening of the glomerular basement membranes; extensive edema and fibroblast proliferation accompanied by a significant infiltration of lymphocytes; numerous renal tubules show indistinct structures, cellular vacuolar degeneration, and varying-sized round vacuoles within the cytoplasm; there is a small amount of lymphocyte infiltration in the interstitium; scattered areas exhibit slight cell necrosis and nuclear fragmentation; small areas of hemorrhage are visible (Figure 7C). Cardiac muscle fibers are uniformly stained, with clear cell boundaries, consistent striations, distinct cross-striations in cardiac muscle cells, and no abnormalities in the interstitium (Figure 7D). The brain did not exhibit significant lesions (Figure 7E). The small intestine features villi on its surface and is covered by a single layer of columnar epithelial cells; there is extensive necrosis and shedding of mucosal epithelial cells; goblet cells are distributed among epithelial cells; the mucosal muscle layer, consisting of double-layered smooth muscle cells, separates the crypts from the submucosa; the submucosa is composed of connective tissue; the remaining layers of the intestinal wall include a muscle layer composed of smooth muscle cells and a serosal layer; there is significant hypertrophy of the muscle layer with an increased number of muscle cells (Figure 7F). These strains showcased distinct pathological changes including extensive tissues phagocytosis in major target organs.

## 4 Discussion

Except for a virulent strain of GAstV that can cause atypical jaundice symptoms and gosling gout, the majority of isolated GAstV strains in Sichuan primarily exhibit the pathological characteristic of inducing severe gout and fatality in goslings. These strains belong to the typical features of GAstV genotype-2. Gout in poultry, also referred to as urate depositions or poultry urolithiasis, denotes the accumulation of excessive urate in the bloodstream, resulting in hyperuricemia that cannot be efficiently eliminated from the body (Xi et al., 2020; Wang Z. et al., 2022). There are two primary factors contributing to gout: excessive presence of uric acid in the body and disrupted host uric acid excretion (Bidin et al., 2012a,b; Wang et al., 2020; Dalbeth et al., 2021). The key driver of heightened uric acid production is the consumption of protein-rich feed containing nucleoproteins and purine bases, such as animal viscera, fish meals, soybeans, and other ingredients (Liao et al., 2015; Wan et al., 2019; Ren et al., 2022). Elevated protein intake, particularly nucleoproteins, contributes to the gradual formation of increased ammonia levels within the body (Xi et al., 2022). If the rate of urate production surpasses the excretion capacity of the urinary organs, it can result in uric acidemia (Xu et al., 2023). Some studies have demonstrated that a highly protein-rich diet for goslings can lead to crystal formation and gout (An et al., 2020). In cases where inflammation and obstruction occur in the kidney or ureter, uric acid excretion becomes obstructed, causing uric acid to accumulate in the bloodstream (Yuan et al., 2018). Uric acid salts precipitate on the surfaces of various organs, including the pleuras, pericardiums, peritoneums, mesenteries livers, kidneys, spleens, and intestines (Zhang X. et al., 2018). A shared characteristic of infectious factors is their potential to induce nephritis, kidney damage (Huang et al., 2021) and hindered uric acid excretion (Wu et al., 2021). These infectious factors encompass the infectious bronchitis virus, avian adenovirus inflammation, egg drop syndrome-76 (EDS-76), renal cryptosporidiosis, and most recently, GAstV.

Among the various viruses originating in geese that have emerged in Sichuan, GAstV comprises the largest proportion, accounting for 85.8% of cases. The isolated strains exhibit the closest genetic similarity to typical strains like G418, AHAU5, JX01, and others. This suggests that the GAstV outbreak in Sichuan likely originated from southeastern coastal areas of China, possibly spreading through horizontal transmission among goslings into the densely populated goose areas in Sichuan. In accordance with the International Committee on Taxonomy of Viruses (ICTV) classification, the categorization of genera is typically based on the p-distance between the complete amino acid sequences of the host and the structural protein. Viruses with a p-distance greater than 75% in the complete ORF2 protein sequence are typically classified as members of the same genotype. Through phylogenetic analysis, 12 isolated GAstV strains are classified as GAstV genotype-2 (G-2), significantly distant from strains belonging to GAstV genotype-1 (G-1). A thorough alignment of multiple sequences revealed substantial amino acid mutations in specific regions of the proteins encoded by these strains (Li et al., 2022). These mutations predominantly occur in the ORF2 genes, resulting in a significant elevation in blood uric acid levels (Liu et al., 2018). Bayesian analysis indicates that GAstV likely emerged in April 1985. The average phylogenetic rate of GAstV has been estimated to be approximately 1.42 × 10^−3^ nucleotide substitutions per site per year, suggesting a relatively slow evolutionary pace (Li et al., 2021).

In summary, the research on GAstV genotype-2 offers valuable insights into the pathological characteristics, epidemiology, genetic evolution, growth kinetics, virulence, transmissibility and pathogenicity. (1) Our research found that GAstV genotype-2 consistently exhibit the distinctive trait of depositing significant amounts of white urate in the viscera and joints. (2) The occurrence of GAstV outbreaks is concentrated in specific cities, notably, Deyang (61.9%), Chengdu (26.8%), followed by Meishan (6.2%), Zigong (4.1%), and Mianyang (1.0%). (3) GAstV infection in Sichuan primarily manifest as single GAstV infection, followed by double and (4) quadruple infection. GAstV typically coinfects with gosling plague viruses, goose reoviruses, adenoviruses, tembusu viruses, and avian influenza viruses. (5) Through analysis of conserved ORF1b genes and highly variable ORF2 genes, it has been established that all isolated GAstV strains prevalent in Sichuan belong to GAstV genotype-2, the earliest identified GAstV strains in Sichuan dates back to 2019. (6) Moreover, it is estimated that these prevalent GAstV strains have undergone evolutionary changes, characterized by an average substitution rate of 4.97 × 10^−2^ nucleotide substitutions. (7) Within this context, GAstV genotype-2 demonstrate varying degrees of virulence, ranging from highly virulent to weak. Each strain also showcases diverse replication situation to susceptible LMH cells, goose embryos, duck embryos, and goslings. Animal experiments underscore the varied virulence of different strains. Highly virulent strains can lead to significant mortality among goslings and goose embryos, whereas weaker strains exhibit limited lethality in these hosts. However, none of the strains were found to cause fatalities in duck embryos. (8) Despite the varying degrees of virulence, a shared characteristic across all strains is their ability to substantially hinder the growth of goslings. GAstV genotype-2 concentrated distributed in liver, kidney and spleen, followed by intestine, heart and brain. All GAstV genotype-2 exhibit different adaptations to different hosts and varying degrees of damage to tissues and organs. Overall, the strains with higher viral load have stronger pathogenicity and exhibit higher lethality against waterfowl in animal infection experiments. This comprehensive study can help researchers to better understand the molecular epidemiology and pathogenic mechanism of GAstV genotype-2.

## Data availability statement

The data that support the findings of this study are available from the corresponding author upon reasonable request. Internal gene sequences of goose astroviruses genotype-2 SCD1 ORF1b, SCD1 ORF2, SCD2 ORF1b, SCD2 ORF2, SCD3 ORF1b, SCD3 ORF2, SCG1 ORF1b, SCG1 ORF2, SCD4 ORF1b, SCD4 ORF2, SCG2 ORF1b, SCG2 ORF2, SCG3 ORF1b, SCG3 ORF2, SCX1 ORF1b, SCX1 ORF2, SCM1 ORF1b, SCM1 ORF2, SCD5 ORF1b, SCD5 ORF2, SCZ1 ORF1b, SCZ1 ORF2, SCG4 ORF1b, SCG4 ORF2 can be found under the NCBI GenBank accession No. OR234621, No. OR234624, No. OR234622, No. OR234615, No. OR234607, No. OR234616, No. OR234610, No. OR234626, No. OR234608, No. OR234617, No. OR OR234611, No. OR234619, No. OQ909424, No. OQ909424, No. OR234613, No. OR234625, No. OR234623, No. OR234620, No. OR234609, No. OR234618, No. OR234614, No. OR234627, No. OR234612 and No. OR234628 respectively.

## Ethics statement

The animal studies were approved by the Institutional Animal Care and Use Committee of Sichuan Agriculture University in Sichuan, China (Protocol Permit Number: SYXK(川)2019-187). The studies were conducted in accordance with the local legislation and institutional requirements. Written informed consent was obtained from the owners for the participation of their animals in this study.

## Author contributions

LX: Conceptualization, Formal analysis, Investigation, Methodology, Software, Writing – original draft. BJ: Conceptualization, Methodology, Software, Validation, Writing – review & editing. YC: Conceptualization, Writing – review & editing. ZG: Conceptualization, Writing – review & editing. YH: Conceptualization, Validation, Writing – review & editing. ZW: Conceptualization, Writing – review & editing. MW: Conceptualization, Validation, Writing – review & editing. RJ: Conceptualization, Writing – review & editing. DZ: Conceptualization, Writing – review & editing. ML: Conceptualization, Validation, Writing – review & editing. XZ: Conceptualization, Writing – review & editing. QY: Conceptualization, Writing – review & editing. YW: Conceptualization, Writing – review & editing. SZ: Conceptualization, Writing – review & editing. JH: Writing – review & editing. XO: Conceptualization, Writing – review & editing. QG: Conceptualization, Writing – review & editing. DS: Conceptualization, Writing – review & editing. AC: Conceptualization, Writing – review & editing. SC: Conceptualization, Data curation, Funding acquisition, Methodology, Project administration, Resources, Supervision, Validation, Visualization, Writing – review & editing.
